# Dark Side of Cancer Therapy: Cancer Treatment-Induced Cardiopulmonary Inflammation, Fibrosis, and Immune Modulation

**DOI:** 10.3390/ijms221810126

**Published:** 2021-09-19

**Authors:** Ettickan Boopathi, Chellappagounder Thangavel

**Affiliations:** 1 Center for Translational Medicine, Department of Medicine, Thomas Jefferson University, Philadelphia, PA 19107, USA; 2 Department of Dermatology and Cutaneous Biology, Thomas Jefferson University, Philadelphia, PA 19107, USA

**Keywords:** chemoradiation, immunotherapy, reactive oxygen species, antioxidants, fibroblast, fibrosis, inflammation, immunity, programmed death ligand-1, cardiopulmonary inflammation

## Abstract

Advancements in cancer therapy increased the cancer free survival rates and reduced the malignant related deaths. Therapeutic options for patients with thoracic cancers include surgical intervention and the application of chemotherapy with ionizing radiation. Despite these advances, cancer therapy-related cardiopulmonary dysfunction (CTRCPD) is one of the most undesirable side effects of cancer therapy and leads to limitations to cancer treatment. Chemoradiation therapy or immunotherapy promote acute and chronic cardiopulmonary damage by inducing reactive oxygen species, DNA damage, inflammation, fibrosis, deregulation of cellular immunity, cardiopulmonary failure, and non-malignant related deaths among cancer-free patients who received cancer therapy. CTRCPD is a complex entity with multiple factors involved in this pathogenesis. Although the mechanisms of cancer therapy-induced toxicities are multifactorial, damage to the cardiac and pulmonary tissue as well as subsequent fibrosis and organ failure seem to be the underlying events. The available biomarkers and treatment options are not sufficient and efficient to detect cancer therapy-induced early asymptomatic cell fate cardiopulmonary toxicity. Therefore, application of cutting-edge multi-omics technology, such us whole-exome sequencing, DNA methylation, whole-genome sequencing, metabolomics, protein mass spectrometry and single cell transcriptomics, and 10 X spatial genomics, are warranted to identify early and late toxicity, inflammation-induced carcinogenesis response biomarkers, and cancer relapse response biomarkers. In this review, we summarize the current state of knowledge on cancer therapy-induced cardiopulmonary complications and our current understanding of the pathological and molecular consequences of cancer therapy-induced cardiopulmonary fibrosis, inflammation, immune suppression, and tumor recurrence, and possible treatment options for cancer therapy-induced cardiopulmonary toxicity.

## 1. Introduction

Thoracic cancers include thymic and windpipe (tracheal) cancers, breast cancer-induced lung metastasis, and non-small cell lung cancers (NSCLCs) as the most common [1]. The cancer may be primary or may originate from other sites such as the prostate, breast [2,3], and pancreatic cancers cells [4], and migrates to the thoracic region and causes lung cancer. Treatment options depend on the stage and type of cancer, which includes surgical removal of cancer tissue followed by chemotherapy or radiation, or a combination of chemoradiation therapy (CRT) and immunotherapy. CRT is the preferred treatment option for thoracic malignancies; however, while it can increase cancer-free survival, it parallelly promotes acute and chronic cardiopulmonary toxicities. The lungs are one of the most sensitive tissues to ionizing radiation and this sensitivity limits the success of radiotherapy for lung cancer treatment. CRT causes early toxicity, occurring within hours to a few days after exposure, and late toxicity, occurring months after the treatment which includes cardiopulmonary tissue fibrosis, inflammation, and alteration of cellular immunity [5,6,7,8,9,10]. Radiation therapy promotes immune suppression via immune modulator programmed death ligand-1 (PD-L1) in cancer patients. These toxicities lead to cardiac myopathy, hypertension, myocarditis, left ventricular dysfunction, ventricular arrhythmias, pulmonary edema, pulmonary hemorrhage, and pulmonary pneumonitis [5,11,12,13,14,15,16,17,18,19,20,21,22].

In this review, we summarize the current state of knowledge on the cardiopulmonary complications of cancer therapy and our current understanding of the pathological and molecular consequences of cancer therapy-induced cardiopulmonary toxicity, as well as the relationship between chronic inflammation and cancer. Different ways by which cancer therapy promotes lung and heart toxicity include 1) cancer therapy-induced cardiopulmonary inflammation and fibrosis, and 2) cancer therapy (radiation therapy)-induced immune suppression. Additionally, in this review, we summarize how the activated macrophages maintain the ECM remodeling during fibrosis and present how chemoradiation-induced cardiopulmonary inflammation promotes carcinogenesis and cancer recurrence. Lastly, we discuss the possible treatment options and management of cancer therapy-induced cardiopulmonary toxicity and chronic inflammation.

## 2. Cancer Therapy-Induced Inflammation and Cardiopulmonary Fibrosis

**Figure 1:**
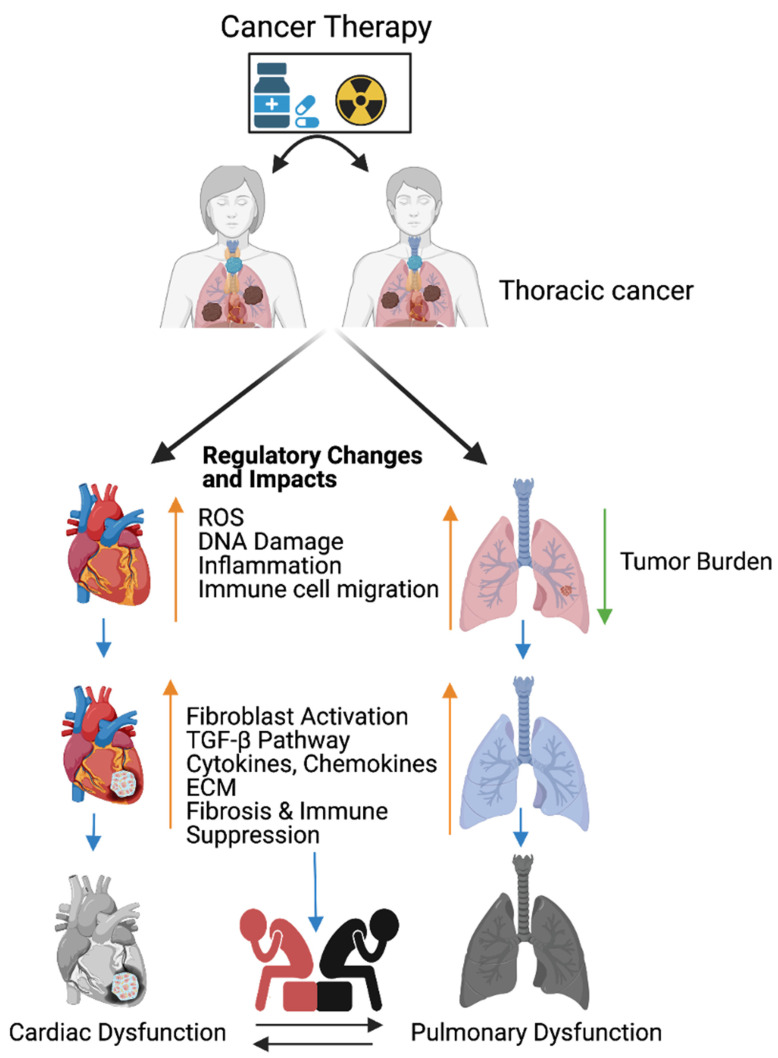
Cancer therapy-induced cardiopulmonary failure. Cartoon illustrates the combination of chemotherapy and ionizing radiation promotes reactive oxygen species (ROS) in thoracic cancer (lung) patients. ROS promotes oxidative stress, DNA damage, immune cell migration, and inflammation, and reduces lung tumor burden. Immune cells at wounded sites secret cytokines, chemokines, and growth factors in cardiopulmonary tissue. Cytokines, chemokines, and growth factors activate myofibroblasts, promote the TGFB pathway, and are involved in the accumulation of the extracellular matrix (ECM). Accumulation of excess ECM induces cardiopulmonary fibrosis, tissue remodeling, immune escape, and cardiopulmonary failure in cancer-free male and female patients who received cancer therapy (chemoradiation therapy). Up arrow indicates the indicated process and the down arrow indicates the reduced tumor burden. This graphic/cartoon is created with BioRender.com agreement # IP22YQRECH.

Lung cancer or thoracic cancers are frequently managed with immunotherapy, radiation, chemotherapy as monotherapy, or radiation combined with chemotherapeutic agents, such as platinum (cisplatin and carboplatin), microtubule inhibitors taxanes (docetaxel and paclitaxel), blood vessel formation inhibitors (bevacizumab), epidermal growth factor inhibitors (erlotinib and necitumumab), and immunotherapies [23] (nivolumab, pembrolizumab, atezolizumab, and ipilimumab). Cancer therapeutics cause cardiopulmonary toxicities including hypertension and myocardial ischemia [23,24,25,26,27,28,29,30], and this is accepted by the American Cancer Society and American Heart Association. According to the US National Cancer Institute (NCI) and based on our previous knowledge, chemotherapy or immunotherapy promote toxicity/inflammation in non-target organs and these inflammations invite migration of neutrophils, macrophages, and cytotoxic lymphocytes to injured and non-injured sites [6]. These immune cells secret cytotoxic substances, proinflammatory cytokines, chemokines, and growth factors that lead to chronic inflammation [31] (Figure 1).

Cancer therapy promotes reactive oxygen species (ROS) such as superoxide and hydrogen peroxide production [32], and these events account for most of the total tissue damage inflicted. ROS also causes mitochondrial DNA (mtDNA) and nuclear DNA damage. Mitochondrial DNA is more sensitive to damage than nuclear DNA due to the lack of repair mechanism response factors within mitochondria. Pro-inflammatory molecules from mitochondria (DNA damage-associated molecular patterns (DAMPs)) enter into the cytoplasm of cells, activate intercellular space receptors, and trigger the recruitment of immune effector cells such as cytotoxic lymphocytes, macrophages, neutrophils, and leukocytes into the damaged tissue site following the death signal [33]. However, chronic damage releases excessive DAMPs [34] that provoke inflammation and immune-related diseases in non-target organs [33] (Figure 1). Additionally, cytotoxic lymphocytes and macrophages arrive to the wound area by transmigration following the neutrophils to clear unwanted cells [35] The inflammatory cells secrete cytokines, chemokines, and growth factors TNF-α and TGF-β; activate fibroblasts; and facilitate the interaction of endothelial, fibroblasts, and macrophages. The immune stimuli recruit more macrophages and macrophages promote fibroblast growth by secreting growth factors such as TNF-α and TGF-β [36], which are responsible for fibrosis.

The consumption of oxygen is needed for the activation of immune cells to migrate to the hypoxic tissue [31] for the purpose of clearing unwanted cells. Hypoxia promotes the generation of ROS and upregulates both TGF-β-signaling and extracellular matrix formation-ECM (collagen deposition) in lung tissue [37]. Additionally, hypoxia promotes VEGF and lactase dehydrogenase expression via the hypoxia inducible factor, HIF*1*-α and HIF-2α. Furthermore, hypoxia promotes fibrogenesis via *SMAD3*-signaling [38]. All these events are accounted for in lung cancer (radiation) therapy led to radiation-induced pneumonitis [31]. To mitigate the radiation damage, patients are administrated with dexamethasone for four weeks after radiation treatment, as dexamethasone decreases the cytokine expression and both *TGF-β* and inflammatory cell infiltration improves the survival rate [31].

### Cancer Therapy Response Factors Promote Cardiopulmonary Toxicity via NFκB-Signaling

Cardiopulmonary dysfunctions caused by cancer therapy limit cancer treatment efficacy and some of the cancer therapeutics such as cisplatin treatment promote oxidative stress, mitochondrial damage, hypertension, and cardiomyopathy approximately 10 years post-treatment [39,40]. Lung cancer patients treated with microtubule inhibitors (taxanes) are at risk for developing hypertension and myocardial ischemia [41,42]. Cisplatin treatment promotes inflammation response factor TNF-α in addition to ROS, which in turn activates the NFκB-signaling responsible for apoptosis and pro-survival genes such as Bcl-2 and fibrosis [43] (Figure 1). NFκB has been implicated in cell survival and immune cell maturation and inflammation, although continuous activation of NFκB functionally impairs the cardiac tissue and promotes heart dysfunction in response injury [44]; worthy to note, though, the underlying mechanism of NFκB-induced heart failure is poorly studied.

## 3. Cancer Therapy (Radiation)-Induced Immune Modulation

**Figure 2:**
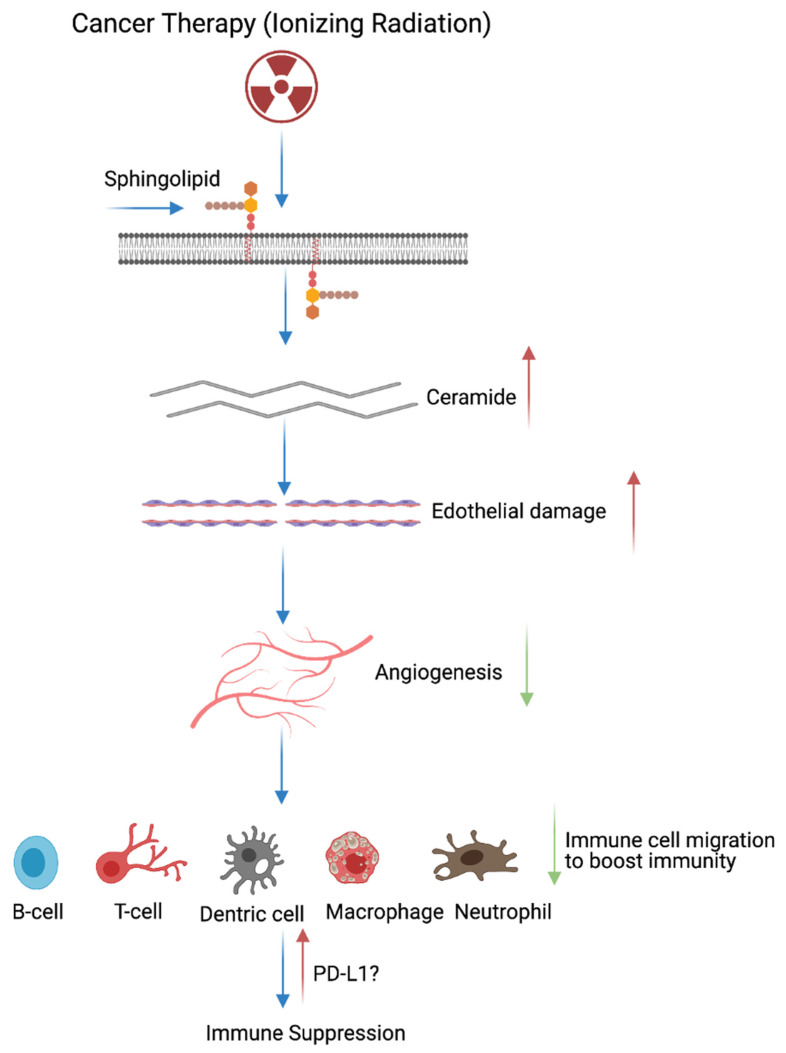
Cancer therapy promotes immune suppression. Cartoon illustrates that radiation therapy directly acts on the plasma membrane bound sphingolipids, cleaves sphingolipids, and generates ceramide through sphingomyelinases. This process leads to the accumulation of ceramide in the injured tissue, causes endothelial cell damage, and inhibits angiogenesis (blood vessel formation). Angiogenesis inhibition restricts the recruitment of immune cells through the bloodstream to the damaged site/inflamed area. Failure in the recruitment of immune cells (neutrophils, macrophages, T cells, B cells, and dendric cells) leads to programmed death ligand-1 accumulation and immune suppression. Note: Up allow indicated the increased process and down arrow indicates inhibited/repressed process. This graphic/cartoon is created with BioRender.com agreement # MJ22YQRUWX.

An average of 50% of cancer patients receive ionizing radiation therapy as a frontline therapy [45]. Ionizing radiation is a major component of the cancer therapy administrated as a single agent or combined with chemotherapy and surgery [46]. The efficacy of the radiation depends on the degree of DNA damage, which results in cancer cell death. Low doses of radiation therapy can stimulate antitumor immunity [47,48] and radiation therapy improves anti-tumoral immunity via three distinct ways: (1) radiation can promote the release of neo-antigens, meaning radiation can improve cancer cell-targeting via CD8+ T cells, and radiation can stimulate chemokines that invite effector T cells; (2) radiation induces the antigen processing machinery and MHC class I cell surface recognition molecules [49,50]; and (3) the radiation response promotes the expression of pro-inflammatory chemo, cytokines, and nature killer cells (NK) [51,52,53,54,55]. Growing evidences suggests that radiation increases the intercellular peptide pool and induces the T cell response to those peptides [50]. However, long-term exposure of radiation and numerous doses can induce immune suppression. Radiation therapy directly acts on the plasma membrane resident sphingolipid and generates ceramide by an enzymatic action [56]. Furthermore, increased ceramide concentrations promote endothelial apoptosis and alter the contraction of cardiomyocytes [57]. Subsequent accumulation of ceramide in the bloodstream further blocks angiogenesis, which leads to low oxygen supply. Deficiency in blood vessels reduce the migration of immune cells to the site of damage to clear the inflammation and maintain cardiopulmonary tissue homeostasis. Failure in immune cell infiltration to the wounded site leads to organ-confined or tissue-specific immune suppression via *PD-L1* immune modulation [58,59,60,61] (Figure 2).

### 3.1. Fibroblast Activation and Cardiopulmonary Inflammation in Response to Cancer Therapy

Generally, fibroblast cells are undifferentiated, activated by a variety of signaling molecules such as platelet-derived growth factors (PDGF) and TGF- β, which are differentiated into myofibroblasts [61]. The differentiated myofibroblasts interact with cardiomyocytes and pulmonary tissues to promote the molecular pathogenesis of fibrosis and the production of the extracellular matrix (ECM). The conversion of fibroblasts to myofibroblasts enables the contraction of the ECM to cover the open wound [62]. Fibroblasts play a vital role in the inflammation and immune cell recruitment at injured sites. Additionally, fibroblasts secrete inflammation responsive cytokines and play a role in the inflammation and immune cell recruitment to wounded sites to clear dead cells and scars. Furthermore, fibroblasts produce inflammation responsive cytokines and chemokines, including TNF-α, TGF-β, IL-6 IL-1, CXCL14, CCL21, cytokine IL-7, and cytokine IL-7 [63,64,65,66,67,68], and trigger immune cells to react on wound-residing dead cells. The accumulation of fluids in the wounded area promotes the hypoxic condition. Pulmonary hypoxia activates fibroblasts in the blood vessels and leads to vascular remodeling with pulmonary hypertension, and pulmonary hypertension leads to mortality [69] (Figure 1).

The combination of chemotherapy and radiation therapy is the most effective treatments for breast, esophageal, and lung cancers. However, the combined therapy promotes early non-symptomatic cardiopulmonary fibrosis responsive risk factors, including the platelet-derived growth factor (PDGF), tumor necrosis factor (TNF), TGFβ, interleukins (IL-1, IL-6, and IL-8), and neutrophil infiltration. Additionally, these treatments induce the late cardiopulmonary fibrogenesis responsive transforming growth factor, IL-4, and IL-13 [28,70]. Histopathological results clearly demonstrate that fibrosis areas are rich in myofibroblast cells, inflammatory cells, and extracellular matrix collagen depositions (ECM) in injured cardiopulmonary organs [29,71]. TGFβ-SMAD-signaling is well studied and characterized in fibrosis models [72,73]. Subsequent accumulation of the ECM in the cardiac tissues leads to myocardial fibrosis, deregulation of heart function, and cardiac failure. Similarly, radiotherapy causes pulmonary pneumonitis and pulmonary fibrosis, which lead to pulmonary remodeling, dysfunction, and pulmonary failure [29] (Figure 1).

### 3.2. Activation of M2 Macrophages, Maintenance of Tissue Archistructure, and Extracellular Matrix Remodeling (ECM)

**Figure 3:**
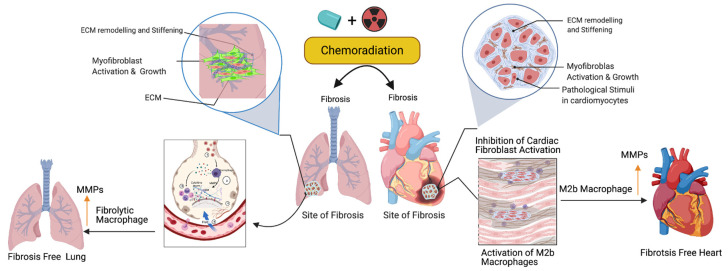
Role of fibrolytic macrophages on the ECM clearance and maintenance of both muscle cell physiology and cellular homeostasis. Following the cancer therapy-induced damage, the immune system triggers the activation of fibrolytic macrophages/M2b macrophages. The activated macrophages migrate to the site of fibrosis and produce or secrete matrix metallopeptidase (MMPs) enzymes to destroy or eliminate excess ECM via phagocytosis/degradation processes, and protect cardiopulmonary muscle cells and their physiological state. Note: Up arrow indicates the upregulation of MMP, M2b macrophages and fibrolytic macrophages. This graphic/cartoon is created with BioRender.com agreement/license # YC22YQS6MB.

Activated fibroblasts (myofibroblasts) have higher migratory behaviors and produce more ECM components than fibroblasts [74], and the net result is the accumulation of excess ECM in the tissue wounds. Macrophages play important roles in cleaning the excess ECM and maintaining tissue skeletal structure. Macrophages are white blood cells and these cells both migrate into the wounded area or tumor and clear cell debris, while unwanted cancer cells and the extracellular matrix are cleared through the phagocytosis mechanism. Macrophages are grouped under M1 (killer) and M2 (repair) categories and they perform anti-inflammatory roles. Macrophages play a key role in muscle repair as well as in regeneration and maintenance [75,76,77]. M2 macrophages migrate to the site of muscle damage, inflammation, and fibrotic tissue (ECM), and macrophages secrete both matrix metalloproteases (MMPs) and lyse unwanted cells. Additionally, macrophages clear the ECM/scars in the wounded area. Fibrosis is an irreversible process at the wounded site with excess ECM deposition. Matrix metalloproteinases are the enzymes involved in the clearing process of excess ECM, target fibrosis, and maintain the cytoskeletal structure of the tissue. To date, 23 MMPs have been reported and most of which are secreted by macrophages [78]. In addition to MMPs, macrophages secrete pro-inflammatory cytokines and chemokines to attract immune cells neutrophils and natural killer cells to wounded sites to destroy the unwanted cells [79]. Fibroblast proliferation is a key event in the fibrosis, as M2b macrophages inhibit fibroblast proliferation responsive proteins [80], produce MMPs, clear the ECM, and maintain cardiopulmonary cellular homeostasis (Figure 3). Additionally, the ECM is critical for fibrosis and tumor progression, as the ECM blocks the cancer therapeutics and limits the efficacy of cancer drugs. Macrophages are the main source of MMPs and MMPs are capable of destroying the ECM. Currently, developed modified macrophages with the chimeric antigen receptor (CAR) will be an ideal therapeutic to target the ECM and maintain cellular integrity during tissue injury [81].

### 3.3. Biomarker Signatures in Cardiopulmonary Toxicity Following Cancer Therapy

Recent advances in omics technology enabled the identification of molecular changes that underlie the development and progression of human diseases, including cancer. The use of multi-omics, which takes the advantage of technologies such as DNA sequencing, exome sequencing, Chromatin immunoprecipitation (ChIP) sequencing, whole-transcriptome analysis, single-cell transcriptome, 10 X visium genomics analysis, proteomics, and metabolomics, has led to the identification of biomarkers in human diseases, including cancer. Oliver et al., 2019, summarized the importance of the multi-omics approach for the identification of biomarkers in the field of oncology [82]. In the field of oncology, genomics and other omics approaches have identified mechanisms in cancer development, treatment resistance, and the recurrence risk, and these findings have been used in clinical oncology to guide treatment decisions. For example, DNA sequencing and whole-transcriptome sequencing of 100 castration-resistant prostate cancer (CRPC) patient samples identified somatic mutations in TET2, DNAMT3B, and BRAF genes [83]. Interestingly, mapping of the E2F1 cistrome using the transcriptomic approach revealed the role of tumor suppressor RB (retinoblastoma protein) in metabolism and cancer.

Several recent studies have used the state-of-the-art mass spectrometry approach to identify cancer-specific biomarkers in human diseases [84,85] and additional analyses of proteomics data led to the identification of the specific pathways involved in mediating carcinogenesis [86]. Wei Chu et al. identified oral cancer specific biomarkers [87] using mass spectrometry in oral cancer patients’ saliva. [88]. Park et al. employed the metabolomics approach (liquid chromatography coupled with mass spectrometry) and identified four biomarkers including L-octanoylcarnitine, 5-oxyproline, hypoxanthine, and docosahexaenoic acid in breast cancer patients’ plasma. L-octanoylcarnitine serves as an early-stage biomarker with 100% positivity for breast cancer [89]. The application of cutting-edge multi-omics technology, such us whole-exome sequencing, DNA methylation, whole-genome sequencing, metabolomics, protein mass spectrometry, single-cell transcriptomics, and 10 X spatial genomics (visium), is warranted to identify early and late toxicity response biomarkers and cancer relapse genes following cancer therapy in cardiopulmonary tissues.

### 3.4. Targeted Therapy-Induced Cardiac Toxicities

One of the recent advancements in cancer therapy is monoclonal antibody-based therapy to target certain molecules to control cancer. Cardiac failure or dysfunction is noted in cancer patients (2–4%) who received anti-PD-1, PD-L1, and bevacizumab (BEV) [90,91], which are monoclonal antibody-based therapies against endothelial cells to target many tumor types; however, this treatment promotes cardiac complications including hypertension, ischemia, and congestive heart failure [92]. The application of the tyrosine kinase inhibitor Erlotinib promotes cardiovascular complications in lung cancer patients [93]. Another monoclonal antibody (necitumumab)-based therapy [94] promotes cardiopulmonary arrest in about 3% of patients. In addition to this, a multi-target tyrosine kinase inhibitor, Nintedanib, targets platelet-derived growth factor (PDGF) receptors and fibroblast growth factors 1, 2, and 3. This agent reduces tumor burden but simultaneously promotes left ventricular dysfunction in cancer patients.

Antioxidants are reductant molecules that react with oxidants and are grouped under two categories, namely endogenous and exogenous. Endogenous antioxidants are produced by the human body, while exogenous antioxidants are produced through the nutrient supply. The exogenous antioxidants fall under the enzymatic and non-enzymatic [95] category. Enzymatic antioxidants possess catalase activity (CAT) that converts hydrogen peroxide to H_2_O_2_ and then to water and oxygen. Glutathione (GSH) is considered the most abundant molecule among the endogenous antioxidants. GSH allows to scavenge ROS either directly or indirectly and it directly reacts with O^−2^ and some other ROS. Aerobic respiration results in increased hydrogen peroxide production, which is detoxified by glutathione peroxide (GPx) by converting two GSH molecules to their oxidized form (GSSG). GSH is recycled by the action of glutathione reductase. Among the antioxidant enzymes, SODs catalyze the dismutation of O^2−^ to H_2_O_2_ and catalase stops the formation of OH by converting H_2_O_2_ to oxygen and water [96]. Nutrients supply non-enzymatic antioxidants, which reduces the oxidative stress-mediated cardiovascular risk [97]. Additionally, natural extracts (polyphenols) are capable of suppressing ROS and inducing the antioxidant defense mechanism [98,99,100]. Some of the commonly used antioxidants are Vitamin E, vitamin C, polyphenols, non-flavonoids, carotenoids, selenium, lipoic acids, coenzymes Q10 [95]. Earlier studies have shown that CTR increases ROS levels and enhances DNA damage, while CTR-induced cardiopulmonary toxicity is associated with increased ROS levels [18,101,102,103,104,105]. Targeting DNA damage caused by ROS is another promising strategy to reduce chemoradiation side effects. Similar to the prophylactic effect of berberine against rat colon carcinoma, previous studies in lungs demonstrated that ROS scavenging at 20 mg/kg once a day for 6 weeks reduces the inflammation of CTR-induced lung toxicity [31,106]. Furthermore, the systemic administration of Tempol (275 mg/kg) in mice exposed to whole-body irradiation showed the radioprotective effect [107,108,109]. Cisplatin has been used for the treatment of cervical, testicular, esophageal, ovarian, bladder, and lung cancers, and this drug has been associated with cardiopulmonary toxicity [7,110,111,112].

### 3.5. Possible Antioxidant Treatments and Prevention of Cancer Therapy-Induced Toxicity

**Figure 4:**
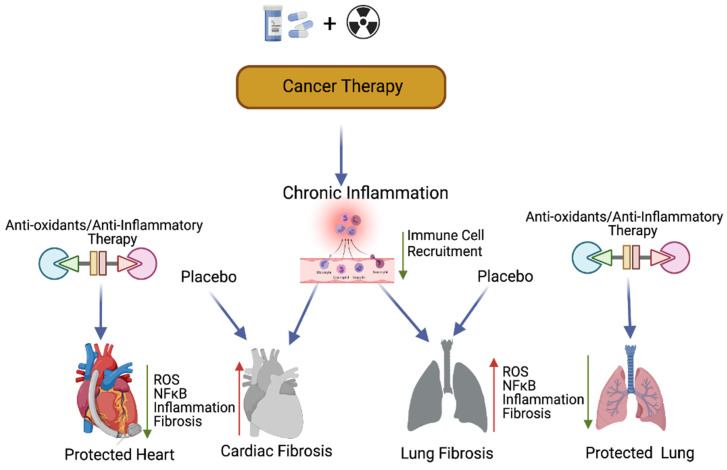
Antioxidants and protection of cancer therapy-induced cardiopulmonary toxicity. This cartoon presentation shows the chemoradiation therapy-promoted cardiopulmonary inflammation, fibrosis via myofibroblast activation, and the NFκB-mediated inflammatory (stress) signaling pathway. The application of antioxidants and inflammatory inhibitors, along with cancer therapy, could protect cardiopulmonary tissue by inhibiting ROS and NFκB-mediated stress signaling. Up arrow indicates the increased levels of ROS, NFκB, inflammation and fibrosis and the down arrow indicates the decreased levels of ROS, NFκB, inflammation, fibrosis and decreased immune cell recruitment. This graphic/cartoon is created with BioRender.com agreement # LD22YQSGUH.

Previous studies have suggested that compounds with antioxidant properties, such as resveratrol [109] and alpha-lipoic acid [113], attenuate cisplatin-induced cardiotoxicities. Antioxidants produced by the human body or consumed from plant products are capable of interacting with and neutralizing ROS, which is induced during oxidative damage/chemoradiation treatment [114,115]. Antioxidants play a key role in controlling cardiopulmonary inflammation and fibrosis [116]. Plant-derived natural antioxidant vitamin A, C, and E from fruits, vegetables, beverages, and cereals [117,118,119,120], and synthetic antioxidant ascorbic acid/vitamin C, glutathione, uric acid, carotenes [121,122,123], and superoxide dismutase (enzyme) are known to suppress oxidative stress [124,125]-induced free radicals (ROS) as well as TGF-β, ERK, and NFκB-mediated signaling, thereby inhibiting fibrogenesis [126] (Figure 4).

### 3.6. Cell Fat Function and Clinical Biomarker Determination

Cancer therapy-induced early cardiopulmonary toxicities are subclinical and not easy to detect in advance. Patients show late symptomatic cancer therapy-induced cardiopulmonary toxicities after 5–10 years post-treatment, which are irreversible. The heart and lung are composed of the heterogenous cell populations. The heart myocardium is composed of 1/3 cardiomyocytes (CMs) and 2/3 remaining fibroblasts, mesothelial, myeloid, lymphoid, adipocytes, pericytes, smooth muscles, endothelial, and neuronal and immune cells [127]. The lung is composed of 40 different cell types including epithelial, smooth muscle, endothelial, nerve, hormone-producing, blood, and structural support cells [128]. Every cell type contributes a specific physiological and pathological function; therefore, it is important to define the cell type specific function when exposed to cancer therapy, which helps to establish clinical biomarkers to target the pathogenesis of fibrosis.

### 3.7. Role of Cancer Therapy-Induced Inflammation in Lung Cancer

Chemotherapy is an effective treatment commonly used for various primary tumors and metastatic cancers. In addition to directly destroying cancer cells, chemoradiation has been shown to also induce inflammation with high levels of ROS, IFN-γ, and damage associated proteins (DAMP) [129]. Cardiopulmonary inflammation causes cardiopulmonary tissue damage in response to chemoradiation. In addition, cancer therapy causes genetic alterations (gene mutations due to impaired DNA repair, DNA methylations, and core histone protein modifications) and enhances ROS levels, which leads to chronic inflammation in cardiopulmonary tissues [130,131,132]. Previous studies have demonstrated that radiation exposure leads to senescence and apoptosis of epithelial and endothelial cells, and initiates strong immune response and inflammation in lung tissues [133,134]. Inflammation promotes the epithelial and mesenchymal transition (EMT) in lung cancer cells [135,136]. Previous studies have shown that there were significant changes in the expression of EMT-related proteins in response to cancer therapy in lung cancer cells [137,138]. Many studies indicated that antioxidants reduce ROS levels [139], thereby reducing the inflammation. Anti-inflammatory drugs have been shown to effectively control tumor progression. However, the adverse side effects associated with the different anti-inflammatory drug treatments have limited their full application to cancer therapy [140,141].

Preclinical and clinical studies suggest that there is strong association between chronic inflammation and carcinogenesis [142]. Chronic inflammation is considered to be one of the characteristics of tumor initiation and progression, and chemotherapy-induced chronic inflammation often endows residual cancer cells with resistance and plays a pivot role in promoting therapeutic resistance and cancer progression. The immune system plays major role in maintaining cellular homeostasis, including cell cycle control and tissue remodeling [142]. Diverse populations of leukocytes move to inflammatory areas and produce cytokines, chemokines, and inflammatory metabolites including prostaglandins and leukotrienes.

### 3.8. Mechanisms of Inflammation-Induced Carcinogenesis and Cancer Therapy-Induced Tumor Recurrence

**Figure 5:**
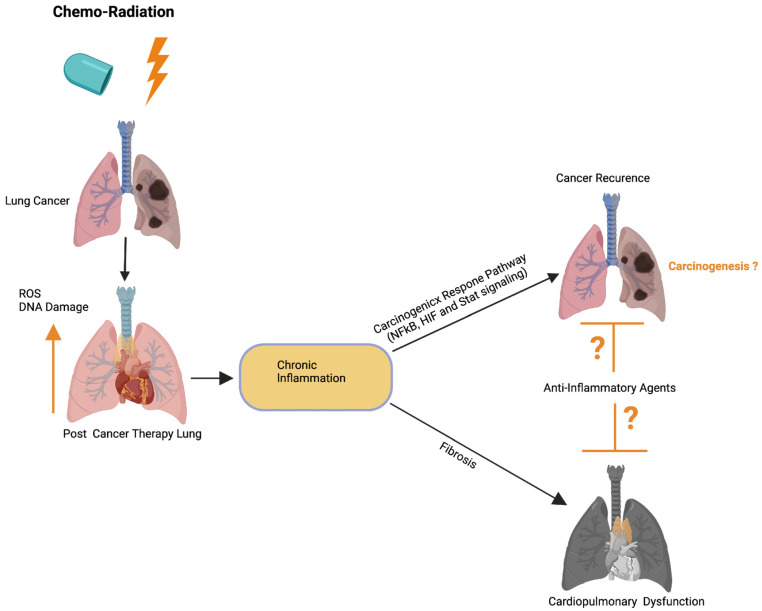
Possible role of cardiopulmonary inflammation on the development of tumor recurrence. Schematics illustrate that chemoradiation therapy inhibits lung tumor burden. Cancer therapy promotes DNA damage and ROS in cardiopulmonary tissue, which leads to chronic cardiopulmonary inflammation. Cardiopulmonary inflammation simultaneously promotes cardiopulmonary fibrosis, organ failure, as well as activation of the carcinogenic response NFkB, HIF, and Stat3 signaling pathways. Possible application of anti-inflammatory therapies could restrict cardiopulmonary fibrosis and tumor recurrence. Up arrow indicates the increased ROS and DNA damage. This graphic/cartoon is created with BioRender.com agreement/license # CU22YQT5TH.

Previous studies have implied that chronic inflammation initiates tumorigenesis through DNA damage, excessive replication, and the inhibition of apoptosis and angiogenesis [143]. Inflammation activates Ras, Myc, and p53-signaling, resulting in mitochondrial impairment and increased ROS production [143]. ROS activates NFkB and STAT3-signaling (Figure 5) [144,145], which cause lung carcinogenesis. Anti-inflammatory drugs have been shown to effectively control tumor progression. Recent therapeutic advancements and meta-analyses suggest that the application of non-steroidal anti-inflammatory drugs (NSAID) inhibits inflammation and lung carcinogenesis [146]. However, the adverse side effects associated with the different anti-inflammatory drug treatments in multiple organs including lung, heart, liver, kidney, the digestive stem, and the brain have limited their full application to cancer therapy [146].

Earlier studies have shown that dying cells (including normal and cancer cells) release growth factors, chemokines, cytokines, interleukins, interferons, and other factors following chemoradiation and these factors prime the carcinogenic-signaling [147]. In addition, dying cells release damage-associated molecular patterns (DAMPs) in response to chemoradiation treatment [148]. DAMPs possibly serve as a ligand for Toll-like receptors (TLRs) expressed on immune cells in the tumor microenvironment (TME) [149]. Cancer therapy not only induces tumor recurrence but also causes drug resistance in patients. Recent study suggests that kinase inhibitors of BRAF, ALK, or EGFR (targeted therapies) promote secretome in melanoma and lung cancer models. RAF and ALK inhibitor-induced secretome increases the proliferation and migration capacity in drug resistance tumor cells [150,151,152,153]. Further studies are warranted to define the mechanism of chemoradiation-induced tumor recurrence and drug resistance in thoracic cancer models.

### 3.9. Role of the Inflammatory Microenvironment in Oncogenesis and Metastasis in Lung Cancer

The correlation between inflammation and cancer was revealed based on the fact that cancer originated in sites of chronic inflammation and the tumor biopsies had abundant inflammatory cells [154]. The chronic dysregulated inflammation has been associated with cancer progression in most malignancies [129,155]. Previous studies have implied that the inflammatory tumor microenvironment (TME) is one of the determining factors for the therapeutic efficacy of radiotherapy, chemotherapy, and immunotherapy [156,157]. The inflammatory tumor microenvironment (TME) plays several roles in tumor progression and metastasis. Chronic overexpression of inflammatory mediators in the TME, as seen in patients with lung cancer [158], may lead to increased oncogenesis, progression, invasion, and metastasis. Radiation therapy-induced pro-inflammatory TME in lung cells are possibly involved in activating interconnected networks of cytokines, adhesion molecules, and damage-associated molecular patterns (DAMPs).

Inflammation promotes EMT through its ability to induce the downregulation of epithelial cell-specific proteins and the subsequent upregulation of mesenchymal cell-specific proteins in lung cancer cells [135,136]. This switch from an epithelial to mesenchymal phenotype underscores the importance of the inflammatory microenvironment in the progression of lung cancer. Previous studies have observed that the majority of patients undergoing conventional radiation therapy for locally advanced lung cancer developed relapses and distant metastasis due to resistance [159]. EMT plays a central role in metastasis [160]. Data from previous studies have shown that there was a significant reduction in E-cadherin expression and a remarkable increase in the expression of both α-SMA and vimentin in response to radiation exposure in lung cancer cells [137,138].

## 4. Conclusions and Future Directions

The increasing population of malignant patients undergoing cancer therapy are suffering from cardiopulmonary dysfunction, cardiopulmonary inflammation, and cancer recurrence following cancer therapy. There are many biomarkers that have demonstrated the ability to predict cardiopulmonary toxicity before the occurrence of clinical signs or symptoms [161]. However, many viable biomarkers do not satisfy the strict criteria, including the solid and easy use of assays, good sensitivity, and specificity. The application of omics technology, such us protein mass spectrometry, metabolomics, whole-genome sequencing, whole-exome sequencing, 10 X spatial visium genomics, and single-cell transcriptomics, is warranted to identify early and late cancer therapy-induced toxicity response biomarkers and tumor relapse biomarkers. Cancer therapy-induced cardiopulmonary inflammation also drives the oncogenesis recurrence and drug resistance in lung cancer patients; however, only a limited amount of evidence is available to support the notion that cancer therapy-induced inflammation promotes oncogenesis and further studies are warranted to prove this concept unambiguously. Patients show late symptomatic cancer therapy-induced cardiopulmonary toxicities after 5–10 years post-treatment, which are irreversible. In order to save patients from therapy-induced non-malignant mortalities, it is warranted to fuse/co-admit chemo-radio protectants such as antioxidants [105] in addition to chemoradiation therapy. Recent advancements in immunotherapy as well as in the application of engineered T cells or CART T cell therapeutic strategies may be possible options to target cardiopulmonary fibrosis [162,163] for the purpose of clearing scar tissues from wounded tissues and maintaining the physiological state of cardiopulmonary cells. It appears that drug-induced cardiopulmonary adverse side effects are genetically determined. Human-induced pluripotent stem cells are best suited for pharmacogenomics research because they are genetically identical to the patients from whom they are derived and can be obtained non-invasively [164,165]. Therefore, pluripotent stem cell technology is highly recommended to investigate the pharmacogenomics of chemotherapy-induced adverse cardiopulmonary effects and this technology potentially could both identify the mechanisms of toxicities and validate the causal genetic variants that contribute to such toxicities.

## Abbreviations

ROS	Reactive Oxygen Species
PD-L1	Reactive Oxygen Species
NSCLC	Non-Small Cell Lung Cancer
TGF-β	Transforming Growth Factor Beta
CTRCPD	Cancer Therapy-Related Cardiopulmonary Dysfunction
SMAD3	Mothers Against Decapendtaplegic Homolog 3
